# The Integrin Signature of Ankylosing Spondylitis: Divergent Regulation of CD29 and CD49b in HLA-B27-Positive Cohorts in Iraqi Patients

**DOI:** 10.22034/ijp.2026.2087485.3651

**Published:** 2026-08-01

**Authors:** Ahmed Ali Alshammari, Muslim Idan Mohsin

**Affiliations:** 1 Department of Pathological Analyses, Faculty of Science, University of Kufa, Kufa, Najaf Governorate, Iraq

**Keywords:** Ankylosing spondylitis, HLA-B27, CD29, CD49b, integrins, PBMCs

## Abstract

**Background & Objective:**

Ankylosing spondylitis (AS) is a systemic inflammatory disorder where the HLA-B27 allele significantly influences disease severity. This study evaluated the molecular profiles of integrins CD29 and CD49b in peripheral blood mononuclear cells (PBMCs) to identify potential biomarkers associated with HLA-B27 status.

**Methods:**

In this cross-sectional study of an Iraqi cohort, 130 participants were recruited: 50 HLA-B27-positive (HLA+) radiographic AS patients, 50 HLA-B27-negative (HLA−) radiographic AS patients, and 30 healthy controls. Transcriptomic and proteomic expressions of CD29 and CD49b were quantified using qPCR and flow cytometry (mean fluorescence intensity, MFI). Hematological and biochemical profiles were also assessed.

**Results:**

CD29 mRNA was significantly upregulated in both patient groups, with a more pronounced median elevation in the HLA+ group (11-fold; P < 0.0001) than in the HLA− group (3-fold; P < 0.001). Surface CD29 protein significantly increased only in the HLA+ cohort (mean MFI approximately 3000; P < 0.0001). Conversely, CD49b mRNA was substantially suppressed, particularly in the HLA+ group (P < 0.0001). Correspondingly, a marked reduction in CD49b surface protein occurred in the HLA+ cohort (mean MFI approximately 300; P < 0.0001), whereas the HLA− group remained statistically comparable to controls. Additionally, the HLA− phenotype was characterized by significant granulocytosis (85%–90%) and more pronounced elevations in urea and ALT levels.

**Conclusion:**

These findings suggest a divergent regulation of CD29 and CD49b integrins associated with HLA-B27 status in Iraqi AS patients. While these integrins may serve as molecular indicators for disease stratification, longitudinal validation is required to confirm their clinical utility as diagnostic or therapeutic targets.

## Introduction

Spondyloarthritis (SpA) encompasses a heterogeneous group of chronic, immune-mediated inflammatory disorders characterized by their predilection for the axial skeleton and sacroiliac joints(1). Clinically, SpA is divided into two primary phenotypes: axial spondyloarthritis (ax-SpA), defined by debilitating spinal stiffness and chronic back pain, and peripheral SpA, which manifests through limb arthritis, enthesitis, or dactylitis. Within the axial spectrum, a critical diagnostic distinction is made based on radiographic progression: ankylosing spondylitis (AS) is characterized by definitive structural damage visible on X-ray, whereas nonradiographic ax-SpA (nr-axSpA) represents an earlier or less progressive stage where such damage remains undetectable(2).

The pathogenesis of AS is a complex, polygenic process with a heritability estimate of approximately 80%(3). The HLA-B27 allele, an MHC class I molecule, remains the most potent genetic risk factor, present in the vast majority of AS patients. However, the incomplete penetrance of this allele suggests that clinical disease is precipitated by environmental triggers in genetically susceptible individuals(4). This interaction is often explained by the “arthritogenic peptide” hypothesis and molecular mimicry, wherein it is hypothesized that the immune system may cross-react with microbial agents, such as Klebsiella pneumoniae, due to structural similarities with self-antigens(5). A pathognomonic hallmark of AS is inflammation at the entheses, the transition zones where tendons and ligaments insert into bone. At these sites, sustained biomechanical stress initiates a cascade of enthesitis and fibrosis, ultimately culminating in pathological bony fusion (ankylosis) and the characteristic “bamboo spine”(6,7).

Epidemiologically, AS typically emerges in late adolescence or early adulthood, exhibiting a significant male predominance in both prevalence and radiographic severity(8). Beyond genetics, lifestyle factors such as cigarette smoking and obesity significantly influence the disease trajectory, exacerbating systemic inflammation and diminishing the efficacy of biologic therapies, particularly anti-TNF agents(9,10). In clinical practice, monitoring disease activity relies on acute-phase reactants (APRs). C-reactive protein (CRP), regulated by IL-6 and TNF-alpha, serves as a sensitive measure of systemic activity, while the erythrocyte sedimentation rate (ESR) remains a standard tool for monitoring inflammatory protein-induced red blood cell aggregation(11,12).

Despite the utility of systemic biomarkers, the persistence of inflammation in AS is fundamentally driven by the recruitment of leukocytes to target tissues, a process tightly regulated by cell adhesion molecules (CAMs)(13). Selectins (L-, P-, and E-selectin) facilitate the initial tethering and “rolling” of immune cells along the vascular endothelium(14). Subsequently, the integrin complex (CD49b/CD29), specifically the α2β1 heterodimer, acts as a critical collagen receptor that mediates cell-matrix adhesion and immune cell trafficking(15). In the context of AS, emerging evidence implicates this complex within the broader framework of the “gut-joint axis,” hypothesizing that it may contribute to the homing of potentially pathogenic Th17 cells toward the collagen-rich environment of the entheses(16,17). Furthermore, it is postulated that CD49b signaling through FAK/Src pathways may promote chronic inflammation and subsequent osteoblast activation, thereby contributing to spinal remodeling(18).

Given that a significant subset of patients remains resistant to conventional NSAIDs and TNF-alpha blockers, there is an urgent need to elucidate the molecular signatures driving leukocyte infiltration. We hypothesize that the mRNA and protein expression levels of specific adhesion molecules (including CD29 and CD49b) in peripheral blood mononuclear cells (PBMCs) are significantly and divergently regulated in AS patients compared to healthy controls. This study aims to quantify these expression profiles using qPCR and protein analysis to establish a baseline molecular signature for AS, potentially identifying novel therapeutic targets and predictive biomarkers for treatment response in the Iraqi population.

## Materials and Methods

### Study Population and Ethical Considerations

This multicenter cross-sectional study was conducted between October 15, 2025, and March 20, 2026. A total of 130 participants (aged 12–85 years) were enrolled and stratified into three cohorts: HLA-B27-positive AS (n = 50): patients meeting the Modified New York Criteria for AS with confirmed HLA-B27 positivity. HLA-B27-negative AS (n = 50): patients with a clinical and radiographic diagnosis of AS but lacking the HLA-B27 allele. Healthy controls (HC, n = 30): age- and sex-matched individuals with no history of autoimmune or inflammatory disorders. Participants were recruited from Marjan Teaching Hospital, Imam Sadiq Hospital, Al-Furat Al-Awsat Hospital, Al-Sadr Medical City, and Al-Hussein Teaching Hospital across the Babil, Najaf, and Karbala governorates in Iraq.

### Inclusion and Exclusion Criteria

Inclusion criteria for AS patients included a confirmed diagnosis based on the Modified New York Criteria and a disease duration of at least 8 months. Exclusion criteria for all participants encompassed the presence of overlapping autoimmune diseases (e.g., rheumatoid arthritis, systemic lupus erythematosus), active or chronic systemic infections, malignancies, and severe hepatic or renal comorbidities. Furthermore, patients receiving biologic disease-modifying antirheumatic drugs (bDMARDs) within the past 3 months or high-dose systemic corticosteroids were excluded to minimize treatment-induced confounding effects on integrin (CD29 and CD49b) expression profiles.

### Sample Size Calculation and Power Analysis

The sample size was determined a priori using G*Power software (version 3.1.9.7). To detect a medium effect size (f = 0.30) in a one-way ANOVA with three independent groups, with an alpha error probability of 0.05 and a statistical power of 80%, a minimum total sample size of 111 participants was required. To account for potential sample degradation during isolation or data loss, the total sample size was increased, and 130 participants were successfully enrolled.

### Clinical Assessment and Disease Severity

Clinical severity and disease activity were objectively evaluated using the Bath Ankylosing Spondylitis Disease Activity Index (BASDAI) and/or the Ankylosing Spondylitis Disease Activity Score (ASDAS). In this study, an “aggressive disease trajectory” or severe clinical status was strictly defined as a BASDAI score ≥ 4.0 or an ASDAS-CRP score ≥ 2.1, reflecting high or very high disease activity.

### Isolation and Cryopreservation of PBMCs

Peripheral blood mononuclear cells (PBMCs) were isolated from whole blood using Ficoll-Hypaque density gradient centrifugation (Solarbio, Beijing, China; Lot No. P4350). Briefly, 2 mL of whole blood was diluted 1:1 with sterile phosphate-buffered saline (PBS). The mixture was carefully layered over 2 mL of Ficoll-Hypaque and centrifuged at 1400 rpm for 40 minutes at room temperature with the brake disengaged. The mononuclear cell layer at the plasma-Ficoll interface was harvested and washed twice in PBS at 300 × g for 10 minutes. The resulting pellet was resuspended in a cryopreservation medium Fetal Bovine Serum FBS supplemented with 5% DMSO). Cell viability and concentration were determined using a Bio-Rad automated cell counter. Samples were adjusted to a final concentration of 2 × 10^6 cells/mL and stored in liquid nitrogen until downstream transcriptomic and proteomic analyses(19).

### RNA Extraction and Quantitative Real-Time PCR (qRT-PCR)

Total RNA was extracted from PBMCs using the Solarbio Life Science RNA Extraction Kit according to the manufacturer’s instructions. RNA purity and concentration were assessed via spectrophotometry. Complementary DNA (cDNA) was synthesized through reverse transcription of stabilized RNA templates.

Quantitative PCR was performed on a GeneLine 7900HT Fast Real-Time PCR System using Primer Design Precision 2× qPCR SYBR Green Master Mix. The relative mRNA expression of CD29 and CD49b was normalized to the housekeeping gene GAPDH using the comparative 2−∆∆Ct method. Validated primer sequences (Macrogen, Seoul, South Korea) are provided in Table 1(20,21).

**Table 1:** Primer sequences used for qRT-PCR.

Gene	Forward Primer (5'—3')	Reverse Primer (5'—3')	(bp)
CD29	CCGCGCGGAAAAGATGAAT	CCACAATTTGGCCCTGCTTG	197
CD49b	GATGCTGTTGCTGTGCCTG	CCCACTAGGAGCCATCGGTT	165
GAPDH	GTCGGAGTCAACGGATTTGG	GACGGTGCCATGGAATTTGC	165

### Flow Cytometric Analysis of CD29 and CD49b

Surface protein expression of CD29 and CD49b was quantified via multicolor flow cytometry. PBMCs were thawed and washed with ice-cold staining buffer (PBS containing 1% BSA and 0.1% sodium azide). Cells were resuspended at 0.5 × 10^6 cells and incubated with conjugated mouse anti-human antibodies: CD29-FITC and CD49b-APC (50 µg/mL) for 45 minutes on ice in the dark.

Following incubation, cells were washed twice (400 × g for 5 minutes) and resuspended in 300 µL of staining buffer. Data were acquired using a URIT BF 730 flow cytometer, collecting a minimum of 10,000 live events per sample. Gating was established based on forward scatter (FSC) and side scatter (SSC) profiles to isolate the lymphocyte and monocyte populations. Data analysis was performed using FlowJo software (version 10.8).

### Statistical Analysis

Statistical processing was performed using GraphPad Prism 11. Data normality was assessed using the Shapiro-Wilk test. Intergroup differences for continuous variables were analyzed using one-way analysis of variance (ANOVA). To correct for multiple comparisons across the three cohorts, ANOVA was strictly followed by Tukey post hoc test. Correlation analyses between integrin molecular markers (CD29, CD49b) and clinical parameters were performed using Pearson (*r*) or Spearman (ρ) coefficients depending on data distribution. To control for the family-wise error rate during multiple correlation testing, a Bonferroni correction was applied where applicable. All correlation results in the text and tables are explicitly presented with their respective correlation coefficients and exact P values. Data are presented as mean ± standard error of the mean (SEM), with P < 0.05 considered statistically significant.

## Results

### Hematological and Biochemical Profiling Stratified by HLA-B27 Status

**Fig. 1:**
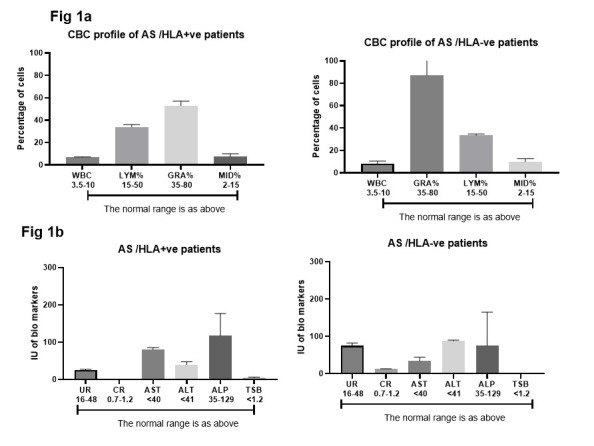
Blood parameters in AS/HLA+ and AS/HLA− patients. (A) Upper panel shows white blood cell count and the percentages of lymphocytes, monocytes, and granulocytes in patients with AS/HLA+ and AS/HLA−. (B) Lower panel shows renal function parameters, including urea and creatinine, and liver function parameters, including GOT, GPT, ALP, and bilirubin. Renal and liver function tests were measured using a Cobas 600 analyzer.

The study cohort comprised 130 participants, including 50 HLA-B27-positive (HLA+) patients, 50 HLA-B27-negative (HLA−) patients, and 30 healthy controls. Complete blood count (CBC) profiles demonstrated distinct patterns between the genetic subgroups (Fig. 1A). Patients in the HLA+ cohort maintained a leukocyte distribution within physiological reference intervals (WBC: 3.5–10 × 10^3/µL; granulocytes [GRA]: 35%–80%). The HLA− cohort exhibited isolated granulocytosis, with granulocyte fractions exceeding the upper normal limit, reaching approximately 85%–90%.

**Fig. 2:**
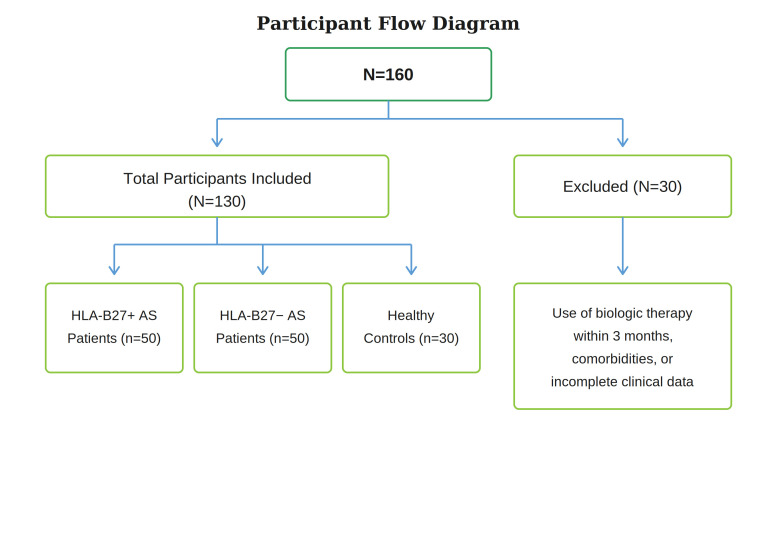
Flow diagram of the study population showing the exclusion of 30 participants (due to biologic therapy within 3 months, comorbidities, or incomplete data) from an initial pool of 160, resulting in a final cohort of 130 individuals divided into HLA-B27+ AS (n = 50), HLA-B27− AS (n = 50), and healthy controls (n = 30).

Biochemical analysis revealed genotype-specific functional profiles (Fig. 1B). Both AS groups demonstrated baseline elevations in specific liver and renal parameters. The HLA− cohort displayed an elevation in urea (UR) and alanine aminotransferase (ALT) levels, frequently surpassing established clinical thresholds (UR: 16–48 mg/dL; ALT: <41 IU/L). Conversely, the HLA+ group was characterized by higher variability and peaks in alkaline phosphatase (ALP) levels. Total serum bilirubin (TSB) and creatinine (CR) remained relatively stable across all groups (Table 2, Fig. 2).

**Table 2:** Clinical and Laboratory Characteristics of Patients with Ankylosing Spondylitis (AS) Stratified by HLA-B27 Status versus Healthy Controls

Characteristic	HLA-B27+ AS (n=50)	HLA-B27- AS (n=50)	HC (n=30)	P-value AS.HLA+/-	P-value AS.HLA+/HC
Age (years)	42.07±12.55	46.07±12.36	40.32±10.34	0.219	0.565
Male	32 (64.0)	28 (43.3)	20 (74.0)	0.003	0.883
Female	18 (36.0)	22 (56.7)	10 (26.0)	—	—
BMI (kg/m2)	29.97±5.65	29.50±5.69	26.11±5.22	0.748	0.009
Hypertension	31 (62.0)	28 (33.3)	13 (46.0)	1.000	<0.001
Diabetes Mellitus	37 (74.0)	23 (46.0)	9 (58.0)	1.000	<0.001
Smoking status, n (%)	36 (72.0)	5 (16.7)	8 (27.0)	0.022	0.500
Hemoglobin (g/dL)	13.37±1.87	13.23±1.52	14.12±1.51	0.752	0.100
WBC count (×109/L)	7.65±2.01	7.29±1.64	6.48±1.32	0.445	0.011
ESR level (mm/h)	21.47±4.16	16.57±3.24	8.11±1.09	0.357	0.004

### Participant Flow Diagram

### Transcriptional and Proteomic Profiling of CD29

**Fig. 3:**
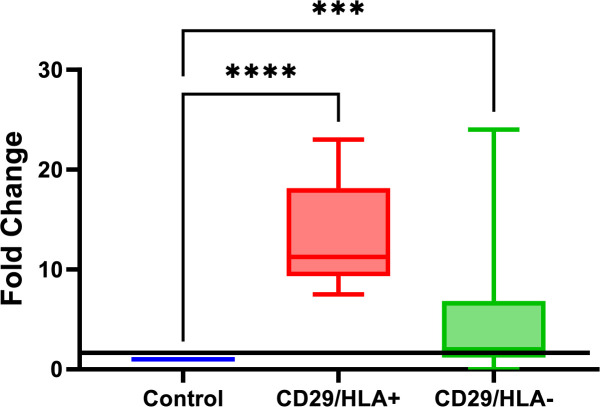
Changes in CD29 mRNA expression in patients with AS/HLA+ and AS/HLA−. CD29 expression in healthy and nonhealthy cases was calculated using the 2−ΔΔCt method following estimation of the housekeeping gene GAPDH. CD29 showed altered expression in PBMCs. The significance of differences was tested by one-way ANOVA, where ***P ≤ 0.001 and ****P ≤ 0.0001 indicate statistical significance.

To evaluate the molecular signature of ankylosing spondylitis (AS) across different genetic backgrounds, quantitative real-time PCR (qPCR) was performed to measure the relative expression of the CD29 (β1 integrin) gene, normalized against the stable housekeeping gene GAPDH. The analysis revealed a statistically significant upregulation of CD29 mRNA in both the HLA-B27-positive (HLA+) and HLA-B27-negative (HLA−) cohorts compared to healthy controls. This upregulation was notably more prominent in HLA-B27-positive patients, with an 11-fold increase (P < 0.0001). The HLA− cohort displayed a significant elevation compared to the baseline (P < 0.001), with a median expression of approximately 3-fold. The HLA− population also exhibited broader distribution and higher variance (Fig. 3).

**Fig. 4:**
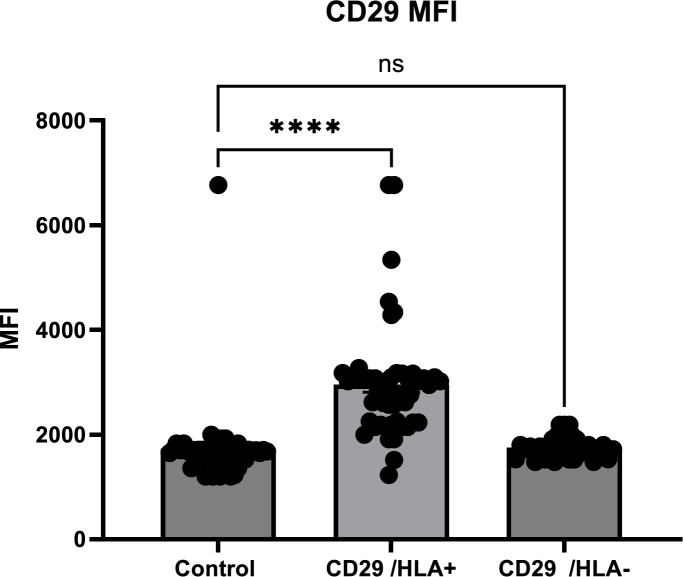
Changes in CD29 protein expression in patients with AS/HLA+ and AS/HLA−. Expression of CD29 in healthy and nonhealthy cases was assessed in PBMCs stained with a fluorescently conjugated antibody specific for CD29. Unstained PBMCs were used as a negative control to determine background fluorescence. The significance of differences was tested by one-way ANOVA, where ****P ≤ 0.0001 indicates statistical significance and ns indicates nonsignificance.

**Fig. 5:**
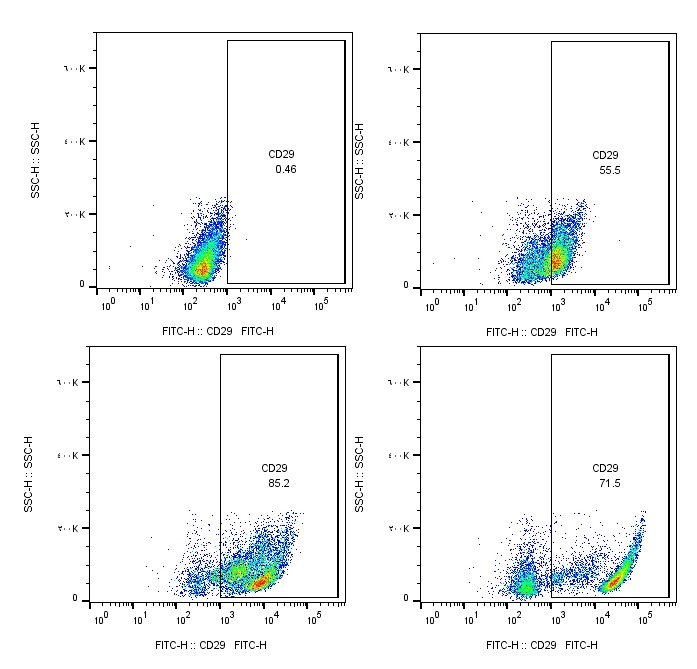
Flow cytometric analysis of CD29 expression in healthy controls and ankylosing spondylitis (AS) patients. PBMCs were stained with a fluorescently conjugated antibody specific for CD29. Unstained PBMCs were used as a negative control to determine background fluorescence. Data were acquired using a URIT flow cytometer and analyzed using FlowJo software. The anti-CD29-FITC antibody was detected using the FITC detector.

Flow cytometry was used to quantify CD29 surface protein expression via mean fluorescence intensity (MFI). Regarding surface protein expression, a significant elevation in MFI for CD29 was specifically identified within the HLA+ cohort, with a mean of approximately 3000 units (P < 0.0001). Several HLA+ subjects presented as high-expression outliers, with MFI values exceeding 6000, suggesting a molecular pattern associated with the patient’s genetic profile. The HLA− cohort did not exhibit a statistically significant increase in surface protein expression compared to the control group (P = ns), with a mean surface MFI centered near 1800 units (Figs. 4 and Fig. 5).

### Transcriptional and Proteomic Profiling of CD49b

**Fig. 6:**
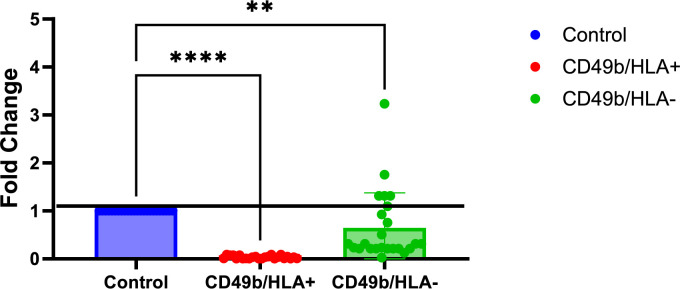
Changes in CD49b mRNA expression in patients with AS/HLA+ and AS/HLA−. CD49b expression in healthy and nonhealthy cases was calculated using the 2−ΔΔCt method following estimation of the housekeeping gene GAPDH. CD49b showed altered expression in PBMCs. The significance of differences was tested by one-way ANOVA, where ***P ≤ 0.001 and ****P ≤ 0.0001 indicate statistical significance.

The transcriptional activity of CD49b (α2 integrin) was analyzed using qPCR and normalized against GAPDH. The results indicated a marked reduction in CD49b mRNA levels across both patient cohorts relative to the healthy control baseline (fold change of 1.0). This extensive suppression was particularly significant in the HLA+ group (P < 0.0001), with fold change values approaching zero, compared to the HLA− group (P < 0.01), with a median expression of approximately 0.3-fold and individual values ranging from near-zero to 3-fold (Fig. 6).

**Fig. 7:**
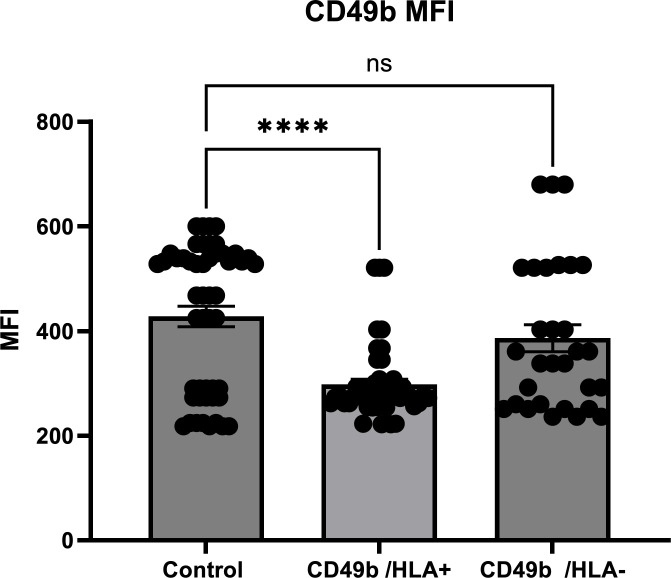
Changes in CD49b protein expression in patients with AS/HLA+ and AS/HLA−. Expression of CD49b in healthy and nonhealthy cases was assessed in PBMCs stained with a fluorescently conjugated antibody specific for CD49b. Unstained PBMCs were used as a negative control to determine background fluorescence. The significance of differences was tested by one-way ANOVA, where ****P ≤ 0.0001 indicates statistical significance and ns indicates nonsignificance.

**Fig. 8:**
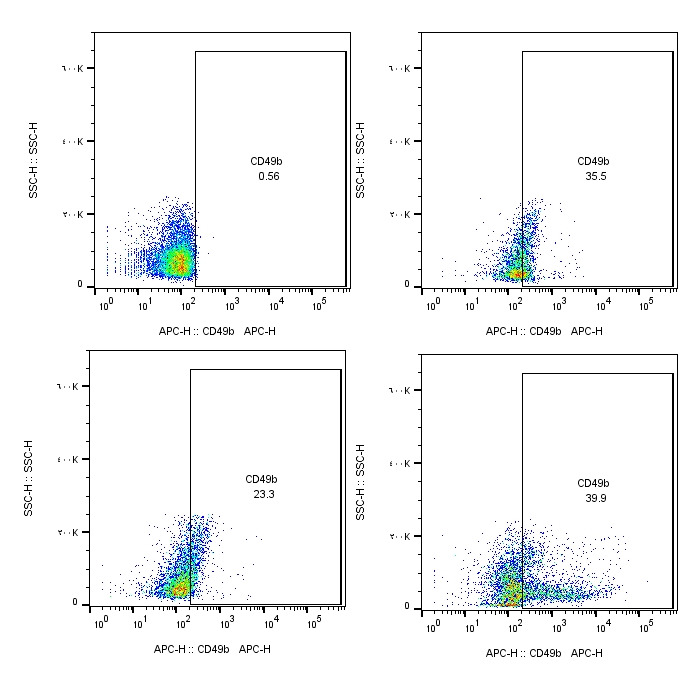
Flow cytometric analysis of CD49b expression in healthy controls and ankylosing spondylitis (AS) patients. PBMCs were stained with a fluorescently conjugated antibody specific for CD49b. Unstained PBMCs were used as a negative control to determine background fluorescence. Data were acquired using a URIT flow cytometer and analyzed using FlowJo software. The anti-CD49b-APC antibody was detected using the APC detector.

Flow cytometric quantification of functional surface receptors via MFI revealed a reduction within the HLA+ cohort, which exhibited a significantly lower mean MFI, approximately 300 units, compared to the healthy control baseline of 430 units (P < 0.0001). The HLA− cohort demonstrated a mean surface MFI of approximately 390 units, which was not statistically different from the controls (P = ns). The HLA− group displayed interindividual variability at the protein level, with specific outliers exceeding 600 units (Figs. 7 and 8).

## Discussion

## Divergent Regulation and Posttran-scriptional Decoupling of CD29

Biologically, the necessity of β1 integrins for leukocyte mechanotransduction and firm arrest to the inflamed vascular endothelium is well established(22). Our transcriptomic findings indicate that while CD29 serves as a significant marker for AS, the quantitative magnitude of its expression appears to be strongly associated with the patient’s underlying genetic profile. This aligns with high-resolution transcriptomic profiling(23), which reported that the HLA-B27 genotype may establish a uniquely aggressive, “primed” chromatin landscape in peripheral leukocytes. Crucially, this genetic stratification offers an alternative perspective to classical clinical models, such as those proposed by(24), which argue that the peripheral systemic inflammatory profile is largely identical between HLA+ and HLA− patients once the clinical threshold for AS is crossed.

Furthermore, the lack of significant protein elevation in the HLA− group, despite elevated mRNA, highlights a critical “decoupling” between mRNA transcription and surface protein translation. This decoupling suggests the potential involvement of complex posttranscriptional regulatory mechanisms or variable protein degradation(25). This is consistent with recent studies showing that inflammatory receptors in less severe clinical phenotypes are frequently subject to tight posttranscriptional silencing, such as microRNA targeting or rapid proteolytic shedding(26,27). Alternatively, this discrepancy might reflect temporal dynamics where mRNA transcription simply precedes stable surface expression, or an increased shedding of the receptor into the soluble fraction (soluble CD29), which is not captured by surface-staining flow cytometry. By demonstrating that elevated CD29 mRNA fails to translate into stable surface protein in HLA− patients, our findings caution against literature that routinely equates elevated peripheral mRNA directly with heightened surface receptor activity(28). Ultimately, significant and stable manifestation of the CD29 protein on the cell surface remains a distinguishing characteristic primarily observed in HLA-B27-positive AS.

## Systemic Downregulation of CD49b and Regulatory Immune Tolerance

CD49b pairs with CD29 to form very late antigen-2 (VLA-2), a primary collagen-binding receptor(22), and serves as a key surface marker for type 1 regulatory T cells (Tr1) responsible for maintaining peripheral immune tolerance(29). The substantial peripheral downregulation of CD49b mRNA in AS starkly contrasts with other inflammatory joint diseases, such as rheumatoid arthritis, where CD49b is heavily upregulated to prepare leukocytes for binding to the collagen-rich synovial matrix(30). This discrepancy indicates that the early transcriptomic pathobiology of AS is characterized by a profound suppression of this marker, which could imply a systemic reduction in circulating regulatory subsets (Tr1) or a targeted receptor downregulation. However, it is also highly plausible that CD49b-expressing cells have preferentially migrated out of the peripheral circulation and localized within the inflamed axial tissues, leading to their apparent depletion in peripheral blood samples.

The extensive transcriptional and proteomic silencing of CD49b in the HLA+ group suggests a severe downregulation of this regulatory machinery. This phenotypic divergence, where significant protein-level reduction is exclusively linked to the HLA-B27-positive genotype, contributes new insights to the ongoing debate in the literature regarding immune tolerance failure in spondyloarthritis. Older studies argued that peripheral regulatory T cells in AS are present in normal quantities but suffer from a functional defect(31). While our MFI data for the HLA− cohort support this functional-only model, the absolute proteomic reduction observed in the HLA+ cohort presents a contrasting perspective. These variations between our findings and earlier reports might stem from differences in patient cohorts, such as disease duration, prior immunomodulatory treatments, or variations in flow cytometric gating strategies.

Instead, the HLA+ data strongly align with state-of-the-art single-cell sequencing studies(32), which argue that severe AS is associated with a quantitative cellular reduction of regulatory subsets. Grouping AS as a single phenotypic entity may mask these genetics-based biochemical divergences. Ultimately, these findings support recent multiomics frameworks demonstrating that the HLA-B27 allele alters the cellular landscape, potentially suppressing specific regulatory genes like CD49b at both the mRNA and stable protein levels(23). While our expression data strongly support this model, future investigations employing direct epigenetic profiling and functional cellular assays are required to definitively confirm these regulatory mechanisms.

## Limitations and Future Research

While this study provides valuable insights, its scope is constrained by an exclusive focus on an Iraqi cohort, meaning these unique genetic, environmental, and cultural dynamics may not fully extrapolate to broader global contexts. Furthermore, the study’s cross-sectional design and lack of longitudinal data preclude definitive causal inferences, while the modest sample size and potential unmeasured confounders warrant caution when interpreting subtle interactions. Ultimately, these boundaries highlight the critical need for future multicenter validation.

## Recommendations for Future Research

To address these limitations and advance the current understanding of these phenomena, future research should transition toward longitudinal designs capable of clarifying definitive causal pathways and tracking progression over time. Methodologically, incorporating comprehensive multivariate models will be essential to systematically account for a wider range of potential confounding variables that may influence outcomes. Furthermore, expanding future cohorts to include larger, more diverse sample sizes will significantly enhance statistical robustness and reduce the margin of error. Finally, conducting comparative, multicenter studies across distinct geographic regions and ethnic backgrounds is critical to validating these findings within a broader global context and establishing their universal applicability.

## Conclusion

In conclusion, this cross-sectional study demonstrates that the molecular profiles of integrins CD29 and CD49b differ significantly based on the HLA-B27 status in patients with ankylosing spondylitis. The elevated expression of CD29 surface protein and the concurrent pronounced downregulation of CD49b in HLA-B27-positive patients point toward a distinct, genetically driven immunoregulatory environment. While our data highlight a strong quantitative association between the HLA-B27 allele and this divergent integrin regulation, the cross-sectional design precludes establishing direct causality or immediate clinical applicability. These findings provide a valuable foundational step, but further longitudinal and mechanistic studies are essential to clarify the precise role of this posttranscriptional decoupling and to determine whether targeting these specific pathways can offer viable diagnostic or personalized therapeutic avenues for severe AS.

## Data Availability

The data that support the findings of this study are available from the corresponding author upon request.

## References

[1] Kocatürk B, Balık Z, Pişiren G, Kalyoncu U, Özmen F, Özen S (2022). Spondyloarthritides: Theories and beyond. Theories and beyond. Front Pediatr.

[2] Poddubnyy D (2020). Classification vs diagnostic criteria: the challenge of diagnosing axial spondyloarthritis. Rheumatology (Oxford).

[3] Inman RD (2021). Axial spondyloarthritis: current advances, future challenges. J Rheum Dis.

[4] Thomas GP, Brown MA (2010). Genetics and genomics of ankylosing spondylitis. Immunol Rev.

[5] Ebringer A, Rashid T, Wilson C, Ptaszynska T, Fielder M (2006). Ankylosing Spondylitis, HLA-B27 and Klebsiella-an overview: proposal for early diagnosis and treatment. Curr Rheumatol Rev.

[6] Duarte JH (2015). Autophagy prevents age-related OA. Nat Rev Rheumatol.

[7] Durmus D, Sarisoy G, Alayli G, Kesmen H, Çetin E, Bilgici A (2015). Psychiatric symptoms in ankylosing spondylitis: their relationship with disease activity, functional capacity, pain and fatigue. Compr Psychiatry.

[8] Jeong H, Bae EK, Hwang J, Park EJ, Lee J, Jeon CH (2021). The effects of sex and estrogen on radiographic progression of ankylosing spondylitis in Korean patients. J Rheum Dis.

[9] Hwang MC, Ridley L, Reveille JD (2021). Ankylosing spondylitis risk factors: a systematic literature review. Clin Rheumatol.

[10] Roberts MJ, Leonard AN, Bishop NC, Moorthy A (2022). Lifestyle modification and inflammation in people with axial spondyloarthropathy-a scoping review. Musculoskeletal Care.

[11] Stanimirovic J, Radovanovic J, Banjac K, Obradovic M, Essack M, Zafirovic S (2022). Role of C‐reactive protein in diabetic inflammation. Mediators Inflamm.

[12] Lapić I, Padoan A, Bozzato D, Plebani M (2020). Erythrocyte sedimentation rate and C-reactive protein in acute inflammation: meta-analysis of diagnostic accuracy studies. Am J Clin Pathol.

[13] Wang W, Yang GJ, Zhang J, Chen C, Jia ZY, Li J (2016). Plasma, urine and ligament tissue metabolite profiling reveals potential biomarkers of ankylosing spondylitis using NMR-based metabolic profiles. Arthritis Res Ther.

[14] Varki A (2025). Origins and Evolution of Essentials of Glycobiology. Glycobiology.

[15] Liu Z, Cai M, Ke H, Deng H, Ye W, Wang T (2023). Fibroblast insights into the pathogenesis of ankylosing spondylitis. J Inflamm Res.

[16] Wei Y, Zhang S, Shao F, Sun Y (2025). Ankylosing spondylitis: from pathogenesis to therapy. Int Immunopharmacol.

[17] Gracey E, Vereecke L, McGovern D, Fröhling M, Schett G, Danese S (2020). Revisiting the gut-joint axis: links between gut inflammation and spondyloarthritis. Nat Rev Rheumatol.

[18] Adorno-Cruz V, Liu H (2019). Regulation and functions of integrin α2 in cell adhesion and disease. Genes Dis.

[19] Mohsin MI, Khudhair MK (2025). TSPAN5: A Potential Biomarker for Methotrexate Resistance in Acute Lymphoblastic Leukemia in Iraq. J Biosci Appl Res.

[20] Kadhim AH, Mohsin MI (2026). Tetraspanin CD9 and CD81: Differential regulation and potential roles in chemoresistance in acute and chronic lymphocytic leukemia. Russ J Immunol.

[21] Mohsin MI, Al-Hilali SAM, Mohsin RI, Mohasin M, Al-Shamarti MJM (2025). IL-8/CD181 Mediated Inflammation in SLE-Associated Hemolytic Anemia. Iran J Pathol.

[22] Hynes RO (1992). Integrins: versatility, modulation, and signaling in cell adhesion. Cell.

[23] Gao Y, Li X, Luo F, Chen R, Zhang X (2025). Integrative mass spectrometry-driven multi-omics and single cell technologies in ankylosing spondylitis: insights into pathogenesis, biomarker discovery, and precision medicine. J Transl Autoimmun.

[24] Rabey M, Smith A, Kent P, Beales D, Slater H, O'Sullivan P (2019). Chronic low back pain is highly individualised: patterns of classification across three unidimensional subgrouping analyses. Scand J Pain.

[25] Vogel C, Marcotte EM (2012). Insights into the regulation of protein abundance from proteomic and transcriptomic analyses. Nat Rev Genet.

[26] Kelly AJ, Long A (2024). Targeting T-cell integrins in autoimmune and inflammatory diseases. Clin Exp Immunol.

[27] Xiangrui X, Yang L, Huijing L (2026). The Th17/Treg axis: a key to understanding and treating autoimmune disorders. Open Life Sci.

[28] Bowness P (2015). HLA-B27. Annu Rev Immunol.

[29] Gagliani N, Magnani CF, Huber S, Gianolini ME, Pala M, Licona-Limon P (2013). Coexpression of CD49b and LAG-3 identifies human and mouse T regulatory type 1 cells. Nat Med.

[30] Brodmann M, Lipp R, Passath A, Seinost G, Pabst E, Pilger E (2004). The role of 2-18F-fluoro-2-deoxy-D-glucose positron emission tomography in the diagnosis of giant cell arteritis of the temporal arteries. Rheumatology (Oxford).

[31] Carrasco E, Gómez de Las Heras MM, Gabandé-Rodríguez E, Desdín-Micó G, Aranda JF, Mittelbrunn M (2022). The role of T cells in age-related diseases. Nat Rev Immunol.

[32] Yao X (2024). scRNA+ TCR-seq revealed the dual TCR pTh17 and Treg T cells involvement in autoimmune response in ankylosing spondylitis. Int Immunopharmacol.

